# Mental health interventions for adolescents in sub-Saharan Africa: A scoping review

**DOI:** 10.3389/fpsyt.2022.937723

**Published:** 2022-08-11

**Authors:** Adam Mabrouk, Gideon Mbithi, Esther Chongwo, Ezra Too, Ahmed Sarki, Mary Namuguzi, Joseph Atukwatse, Derrick Ssewanyana, Amina Abubakar

**Affiliations:** ^1^Institute for Human Development, Aga Khan University, Nairobi, Kenya; ^2^School of Nursing and Midwifery, Aga Khan University, Kampala, Uganda; ^3^Family and Youth Health Initiative (FAYOHI), Dutse, Jigawa State, Nigeria; ^4^Alliance for Human Development, Lunenfeld Tanenbaum Research Institute, Toronto, ON, Canada; ^5^Neurosciences Group, KEMRI-Wellcome Trust Research Programme, Centre for Geographic Medicine Research (Coast), Kilifi, Kenya

**Keywords:** mental health, intervention, adolescents, scoping review, sub-Saharan Africa

## Abstract

**Background:**

Globally, adolescents are vulnerable to mental health problems, particularly those from sub-Saharan Africa (SSA) due to impoverished living conditions and a higher prevalence of chronic conditions such as HIV/AIDS in the region. The COVID-19 pandemic has further exacerbated this risk. This calls for an urgent need for evidence-based adolescent mental health interventions to reduce the risk and burden of mental health problems in SSA. The review aims to identify and characterize existing adolescent mental health interventions in SSA, as well as to evaluate their implementation strategies and effectiveness.

**Methods:**

We systematically searched PubMed, African Index Medicus, PsycINFO, Web of Science, and CINAHL databases for relevant articles. Furthermore, we searched gray literature databases, including Think Tank search, open gray, NGO search engine, and IGO search engine for additional relevant articles. The scoping review was conducted to identify original research articles on mental health interventions among adolescents in sub-Saharan Africa published from database inception to 31 December 2021. We carried out a narrative synthesis to report our findings.

**Results:**

Our literature search generated 4,750 studies, of which 1,141 were duplicates, 3,545 were excluded after screening, and 64 articles met the inclusion criteria. The 64 studies describe a total of 57 unique mental health interventions comprising 40,072 adolescents. The nature of these interventions was diverse, encompassing various implementation strategies such as economic-based, family strengthening, psychoeducation, interpersonal psychotherapy, Cognitive Behavioral Therapy, and resilience training, among others. Most of the interventions were selective interventions that targeted adolescents at high risk of developing mental health problems including adolescents living with HIV, war-affected adolescents, orphans, adolescents from poorer backgrounds, and survivors of sexual violence. Half of the interventions were delivered by lay persons. Sixty-two of the eligible studies examined the effectiveness of the mental health interventions, of which 55 of them reported a positive significant impact on various mental health outcomes.

**Conclusions:**

The review findings show that there exist several diverse interventions that promote mental health among adolescents in sub-Saharan Africa. These interventions can be implemented in diverse settings including schools, communities, health facilities, and camps, and can be delivered by lay persons.

## Background

Approximately 792 million people live with a mental disorder worldwide with many of these cases emerging during adolescence (1, 2). Mental health problems in adolescents have been on the rise in recent years, and they are linked to significant psychosocial, economic, and physical health burdens for affected adolescents, caregivers, and host communities (1, 3, 4). Across the world, mental health problems including, depressive disorders, anxiety disorders, and suicide, affect 10–20% of children and adolescents and account for 16% of the total burden of disease among adolescents aged 10–19 years (5). In Sub-Saharan Africa (SSA), one out of every seven children and adolescents (14.3%) suffers from major psychological problems, with nearly 10% qualifying for a psychiatric diagnosis (6).

The most recent systematic review of studies from Sub-Saharan African countries encompassing a total population of 97 616 adolescents found the following prevalence estimates; 40.8% for emotional and behavioral problems, 29.8% for anxiety disorders (29.8%), 26.9% for depression, 21.5% for PTSD, and 20.8% for suicidal thoughts (7). The prevalence rates reported from the review are high in comparison with studies from other regions (8–10).

The high burden of adolescent mental health morbidity in SSA is contributed by heightened psychosocial stressors such as exposure to chronic poverty, abuse, exposure to violence, and higher prevalence of certain conditions such as HIV/AIDS in the region (7), coupled with the rapid physical, emotional and social changes that occur during this period (11). The COVID-19 pandemic has also placed additional stress on adolescent mental health (12). The negative impacts of poor mental health in adolescents include poor educational performance, poor physical functioning, and unemployment which greatly reduces their overall quality of life (13).

Despite the high burden of mental health problems in adolescents and the associated negative consequences, the mental health of adolescents remains neglected (14). Furthermore, adolescent mental health services remain scarce, particularly in SSA (15). The high burden of mental health problems, and the scarcity of services, necessitate the need for urgent evidence-based mental health interventions to promote mental health among adolescents.

Therefore, the purpose of this scoping review was to identify interventions that have been implemented to address mental health problems among adolescents in SSA. Specifically, the review also aimed to assess the effectiveness of the identified interventions in reducing adolescent mental health problems. Lastly, the review aimed to evaluate the implementation strategies of the interventions.

## Methodology

This scoping review was guided by the methodological framework proposed by Arksey and O'Malley and further advanced by Levac et al. (16, 17). The framework recommends six stages to be followed in conducting scoping reviews. These stages include (i) identifying the research question (ii) identification of the relevant studies (iii) selecting the studies (iv) charting the data (v) collating, summarizing, and reporting the results, and (vi) consultation. We reported our results based on guidelines by the preferred reporting items for systematic reviews and meta-Analysis (PRISMA) statements (19).

### Identification of relevant studies

To capture the full range of mental health interventions targeting adolescents in SSA, all peer-reviewed and non-journal articles (gray literature) were included. The eligibility criteria for including studies and documents in this review was that a component of mental health interventions targeting adolescents in SSA must have been reported. There were no limits on the date of publication or language during the search.

### Search strategy

We systematically searched the following electronic databases: PubMed, African Index Medicus, PsycINFO, Web of Science, and CINAHL (from database inception to 31 December 2021). Additionally, we searched the following gray literature databases: think tank search, open gray, NGO search engine, and IGO search engine. For this review, the electronic literature search followed the three-step search strategy recommended by the Joanna Briggs Institute (18). The first was a preliminary search in a common online database. The search terms “Mental health”, “Intervention” and “adolescents” were used in PubMed. AM, ET, and EC analyzed the keywords used in the titles and abstracts of the identified articles.

In the second step, relevant keywords in the titles and abstracts of the identified papers were reviewed to compile a list of terms to guide in undertaking a detailed literature search in all the databases. Search terms included Medical Subject Headings (MeSH) and keywords related to mental health, mental disorders, intervention, and adolescents.

Based on preliminary searches, we combined the four search terms (“Mental health”, “Intervention”, “Adolescents”, Sub-Saharan Africa”) with the “AND” Boolean operator. Respective synonyms for these search terms were combined using the “OR” Boolean operator (Table 1). The refined final search strategy was developed in consultation with AA, DS, and an experienced librarian and applied to the databases. This was applied to fit the specifications of each database. Results from the individual database searches were retrieved and uploaded to EPPI-Reviewer (Version 4.12.2.0). Lastly, the reference lists of the selected reports and articles were further explored to identify additional studies meeting the eligibility criteria.

**Table 1:** List of search terms used.

**Pubmed**
(((((((((((“mental health”[MeSH Major Topic]) OR (“mental disorders”[MeSH Major Topic])) OR (“internalizing”[All Fields] OR “internalizing and externalizing”[All Fields] OR “internalizing and externalizing psychiatric disorders”[All Fields] OR “internalizing and externalizing behavior”[All Fields])) OR (“behavioral problem”[All Fields])) OR (“behavioral problem”[All Fields])) OR (“depressive symptom”[All Fields])) OR (“depressive disorder”[All Fields])) OR (“mood disorder”[All Fields])) OR (“peer problem”[All Fields])) AND ((((“intervention”[All Fields]) OR (“promotion”[All Fields])) OR (“prevention”[All Fields])) OR (“programs”[All Fields]))) AND (((Adolescen* OR Child* OR young people OR teen* OR youth*) OR (“young men”[All Fields])) OR (“young women”[All Fields]))) AND (“Africa South of the Sahara” OR “Sub Saharan Africa”[tiab] OR “sub-Saharan Africa”[tiab] OR “Sub-Saharan Africa”[tiab] OR “Angola”[tiab] OR “Benin”[tiab] OR “Botswana”[tiab] OR “Burkina Faso”[tiab] OR “Upper Volta”[tiab] OR “Burundi”[tiab] OR “Cameroon”[tiab] OR “Cape Verde”[tiab] OR “Central African Republic”[tiab] OR “Chad”[tiab] OR “Comoros” [tiab] OR “Congo”[tiab] OR “Cote D'ivoire”[tiab] OR “Ivory Coast”[tiab] OR “Zaire”[tiab] OR “Democratic Republic Of The Congo”[tiab] OR “ French Somaliland”[tiab] OR “Djibouti”[tiab] OR “Equatorial Guinea”[tiab] OR “Eritrea”[tiab] OR “Ethiopia”[tiab] OR “Gabonese Republic”[tiab] OR “Gabon”[tiab] OR “Gambia”[tiab] OR “Gold Coast”[tiab] OR “Ghana”[tiab] OR “Guinea”[tiab] OR “Guinea-Bissau”[tiab] OR “Kenya”[tiab] OR “Basutoland”[tiab] OR “Lesotho”[tiab] OR “Liberia”[tiab] OR “Malagasy Republic”[tiab] OR “Madagascar”[tiab] OR “Nyasaland”[tiab] OR “Malawi”[tiab] OR “Mali”[tiab] OR “Mauritania”[tiab] OR “Mauritius”[tiab] OR “Mayotte”[tiab] OR “Mozambique”[tiab] OR “Namibia”[tiab] OR “Niger”[tiab] OR “Nigeria”[tiab] OR “Reunion”[tiab] OR “Rwanda”[tiab] OR “Ruanda-Urundi”[tiab] OR “Sao Tome & Principe”[tiab] OR “Sao Tome”[tiab] OR “Senegal”[tiab] OR “Seychelles”[tiab] OR “Sierra Leone”[tiab] OR “Somalia”[tiab] OR “South Africa”[tiab] OR “South Sudan”[tiab] OR “Sudan”[tiab] OR “Swaziland”[tiab] OR “Eswatini”[tiab] OR “Togolese Republic”[tiab] OR “Togo”[tiab] OR “Uganda”[tiab] OR “United Republic Of Tanzania”[tiab] OR “Tanzania”[tiab] OR “Zambia”[tiab] OR “Zimbabwe”[tiab] OR “Rhodesia”[tiab] OR “Africa Eastern”[tiab] OR “Africa Southern”[tiab] OR “Africa Western”[tiab] OR “Africa Central” [tiab]) Filters: from 1000/1/1 - 2021/12
**African index medicus**
tw:(mental health OR mental disorder OR internalizing OR behavioral symptom OR emotional problem OR behavioral problems OR depressive symptom OR peer problem OR mood disorder OR ptsd) AND (Promotion OR Intervention OR prevention OR program) AND (db:(“AIM”))
**CINHAL**
(mental health OR mental disorders OR internalizing OR internalizing and externalizing OR internalizing and externalizing psychiatric disorders OR internalizing and externalizing behavior OR behavioral problem OR behavioral problem OR depressive symptom OR depressive disorder OR mood disorder OR peer problem) AND (intervention OR promotion OR prevention OR programs) AND (Adolescen* OR Child* OR young people OR teen* OR youth* OR young men OR young women) AND (Africa South of the Sahara OR Sub Saharan Africa OR sub-Saharan Africa OR Sub-Saharan Africa OR sub Sahara OR sub-Sahara OR SSA OR Africa Eastern OR Africa Southern OR Africa Western OR Africa Central)
**Web of science**
(mental health OR mental disorders OR internalizing OR internalizing and externalizing OR internalizing and externalizing psychiatric disorders OR internalizing and externalizing behavior OR behavioral problem OR behavioral problem OR depressive symptom OR depressive disorder OR mood disorder OR peer problem) AND (intervention OR promotion OR prevention OR programs) AND (Adolescen* OR Child* OR young people OR teen* OR youth* OR young men OR young women) AND (Africa South of the Sahara OR Sub Saharan Africa OR sub-Saharan Africa OR Sub-Saharan Africa OR sub Sahara OR sub-Sahara OR SSA OR Africa Eastern OR Africa Southern OR Africa Western OR Africa Central)
**Psych info**
(mental health OR mental disorders OR internalizing OR internalizing and externalizing OR internalizing and externalizing psychiatric disorders OR internalizing and externalizing behavior OR behavioral problem OR behavioral problem OR depressive symptom OR depressive disorder OR mood disorder OR peer problem) AND (intervention OR promotion OR prevention OR programs) AND (Adolescen* OR Child* OR young people OR teen* OR youth* OR young men OR young women) AND (Africa South of the Sahara OR Sub Saharan Africa OR sub-Saharan Africa OR Sub-Saharan Africa OR Angola OR Benin OR Botswana OR Burkina Faso OR Upper Volta OR Burundi OR Cameroon OR Cape Verde OR Central African Republic OR Chad OR Comoros OR Congo OR Cote D'ivoire OR Ivory Coast OR Zaire OR Democratic Republic Of The Congo OR French Somaliland OR Djibouti OR Equatorial Guinea OR Eritrea OR Ethiopia OR Gabonese Republic OR Gabon OR Gambia OR Gold Coast OR Ghana OR Guinea OR Guinea-Bissau OR Kenya OR Basutoland OR Lesotho OR Liberia OR Malagasy Republic OR Madagascar OR Nyasaland OR Malawi OR Mali OR Mauritania OR Mauritius OR Mayotte OR Mozambique OR Namibia OR Niger OR Nigeria OR Reunion OR Rwanda OR Ruanda-Urundi OR Sao Tome & Principe OR Sao Tome OR Senegal OR Seychelles OR Sierra Leone OR Somalia OR South Africa OR South Sudan OR Sudan OR Swaziland OR Eswatini OR Togolese Republic OR Togo OR Uganda OR United Republic Of Tanzania OR Tanzania OR Zambia OR Zimbabwe OR Rhodesia OR Africa Eastern OR Africa Southern OR Africa Western OR Africa)

### Study selection

We used EPPI-Reviewer (Version 4.12.2.0) for managing references of the identified studies and duplicates were automatically removed before commencing the two-stage selection process. Study titles and abstracts were reviewed for eligibility based on the inclusion and exclusion criteria described below. The screening was done independently by two authors (GM & AM) and disagreements were resolved by consensus. The results of the two were reviewed by the team. Full-text articles of selected abstracts were retrieved after which they were also assessed for eligibility by GM and AM.

#### Inclusion criteria

The full inclusion criteria of the review entailed;

a. Study population: studies were included if the reported age range, and/or mean, and/or median age was between 10–19 years.
b. Outcome measures: studies were included if they reported on an intervention that targeted any mental health problem among adolescents, such as externalizing problems (hyperactivity, attention, aggression, and conduct problems), internalizing problems (anxiety, depression, somatization, and withdrawal) and psychological and social wellbeing problems (emotional, hopelessness, self-esteem, self-concept, feelings of isolation, lack of social support and resilience).
c. Geographic location: conducted in Sub-Saharan Africa.
d. Study design: any type of original empirical intervention research such as quantitative studies, including randomized controlled trials, quasi-experimental designs, pre-post evaluations, open trials, and post evaluations, intervention mapping techniques, and mixed-method studies.

#### Exclusion criteria

Exclusion criteria included.

a. Study types: secondary literature (scoping reviews, literature reviews, systematic reviews, and meta-analysis), letters to the editor, protocols, and case series.
b. Outcome measures: studies having interventions that did not target any mental health outcome.

### Charting the data

The data extraction form was developed by AM and piloted by AM and GM who independently extracted the data from a sample of 10% of the included articles. The results of the two reviewers were also compared and discussed by the research team. Data extraction for the remaining included studies was also independently carried out by AM and GM. Where disagreements arose, the two authors re-assessed the specific article and reached a consensus. Subsequently, we updated our search following the initial submission of our article to the journal, in which GM extracted data for additional articles identified and AA confirmed the accuracy of data extraction. We extracted the following information from the included articles: (i) study and sample characteristics (name of the first author, year of publication, country of study, study design, sample size, population targeted (i.e. general adolescent population or specific sub-populations), and age of participants), (ii) Intervention/program characteristics (name of the intervention, type of intervention, who implemented the intervention, study outcomes, delivery method, setting of delivery (i.e. clinic-based, school-based or community-based), duration of intervention, the impact of the intervention (if evaluated), and challenges faced during the implementation of the intervention.

### Collating, summarizing, and reporting the results

To create a summary of the included studies, we collated findings relating to our review questions. We grouped and summarized the identified mental health interventions based on the mental health outcomes they targeted, their setting of delivery, and their impact on adolescent mental health. The results were then reported following the PRISMA extension for scoping reviews (PRISMA-ScR) guidelines (19).

## Results

**Figure 1:**
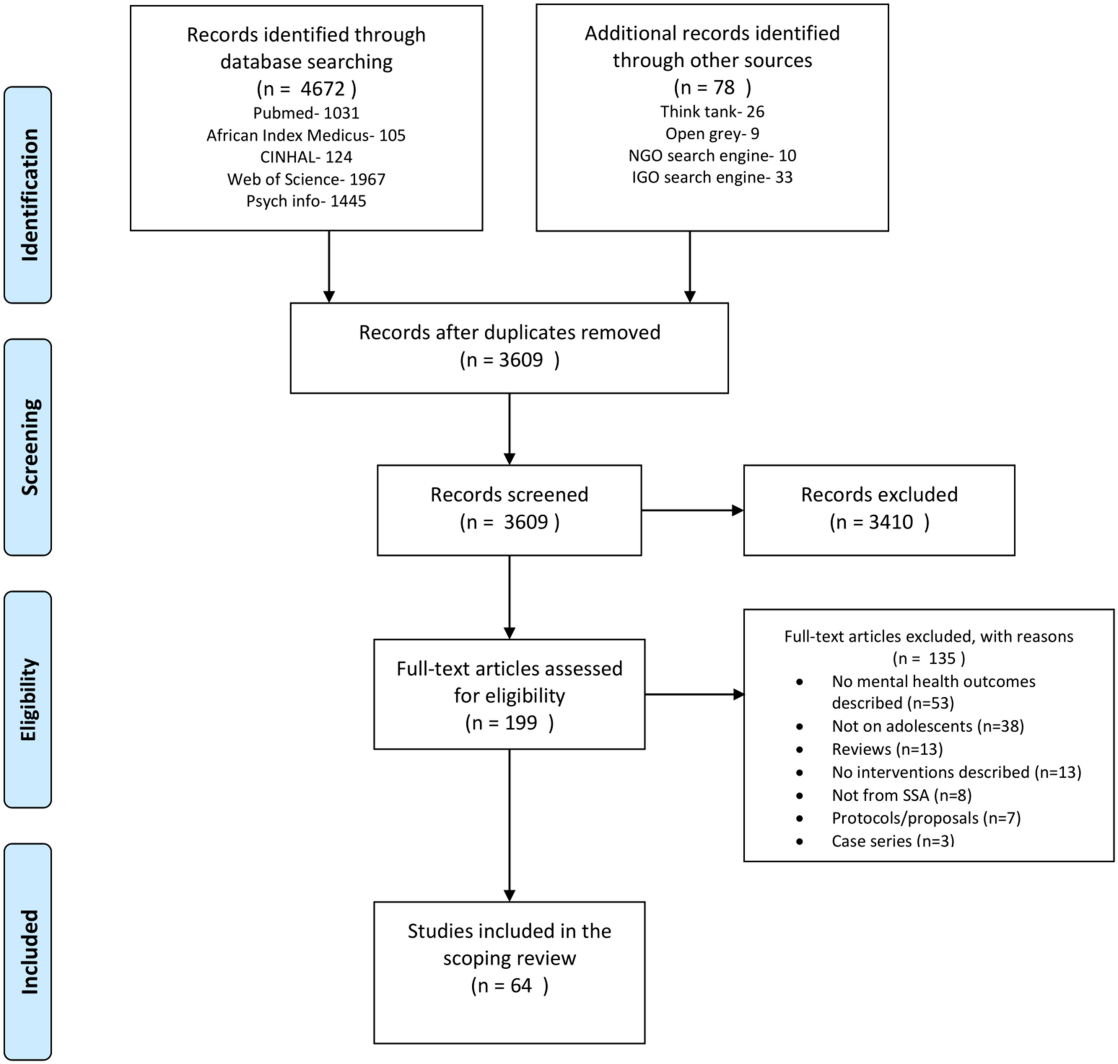
Flowchart showing the scoping review process.

Figure 1 is the PRISMA flow diagram detailing the study selection process. The literature search of the electronic databases yielded 4,672 articles. An extra search in the gray literature yielded an additional 78 articles. After the removal of 1,141 duplicate records, 3,410 articles were found to be unlikely to be relevant after being screened by title and abstract and were thus excluded. After a detailed full-text review of the remaining 199 articles, we excluded 135 studies as they did not meet our inclusion with reasons for the exclusion provided in Figure 1. Although we identified non-English studies in our search, none of them met the inclusion criteria. 64 studies describing 57 mental health interventions among adolescents in SSA were included.

### Study characteristics

**Figure 2:**
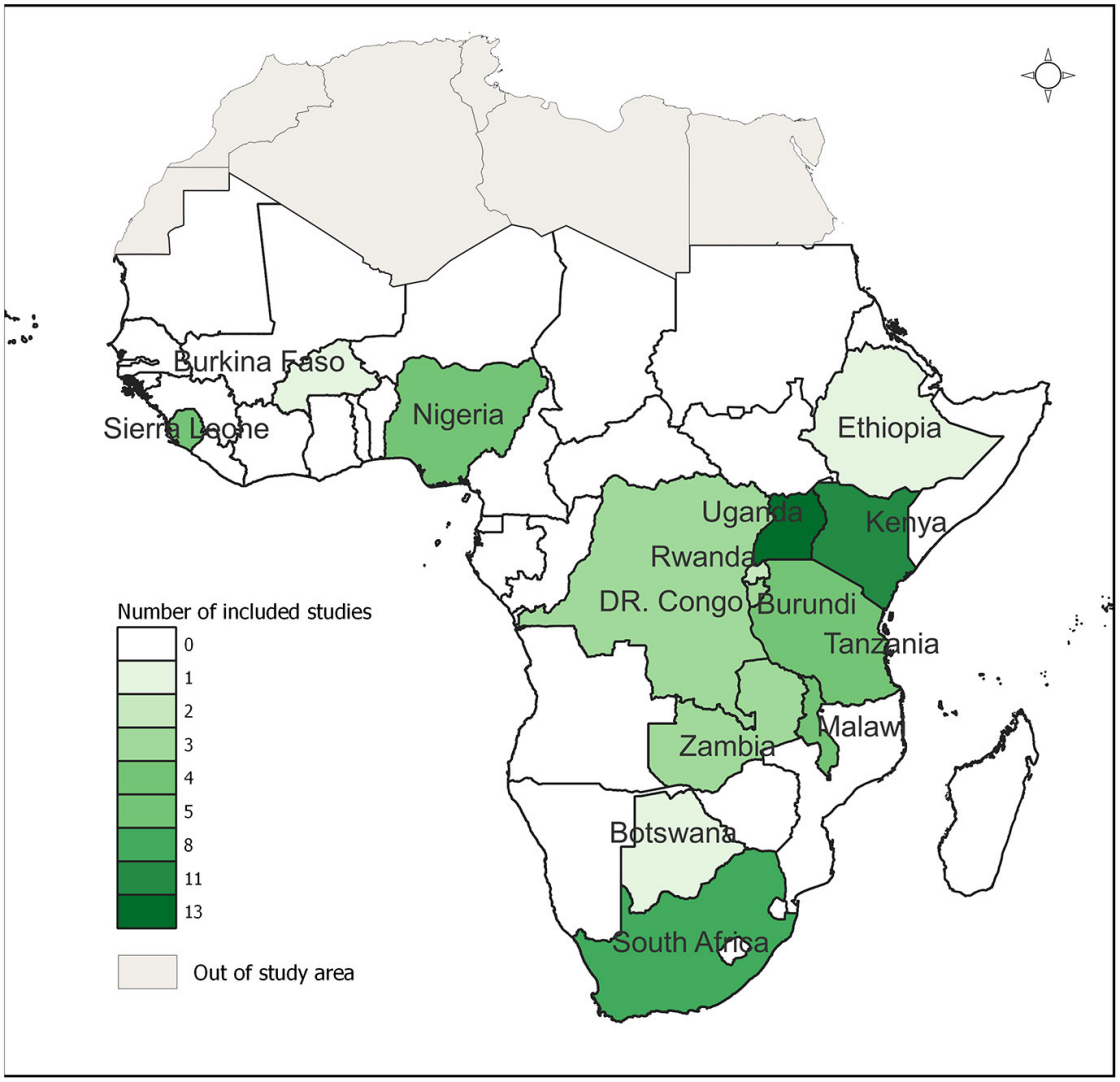
Geographic distribution of the studies included in the scoping review.

The characteristics of the 64 studies and interventions included in this review are shown in Supplementary Table 1. The included studies emanated from 15 countries with more than half (53.1%, *n* = 34) of these studies from only three countries in SSA (Uganda, Kenya, and South Africa). Figure 2 shows the geographical distribution of the studies. A few studies (3.1%, *n* = 2) were multi-country studies.

All the 64 studies were published between 2007 to 2021 with the majority (93.8%, *n* = 60) published over the last decade (2011–2021) while over half (51.6%, *n* = 33) were published in the last 5 years (2017–2021). Only one study was included from the gray literature search (20). In terms of study design, most of the studies (67.2%, *n* = 43) were randomized controlled trials. Five studies incorporated a mixed-methods approach (20–24) while one study used a qualitative intervention mapping technique (25). The remaining studies were either single-arm trials (15.6%, *n* = 10) or quasi-experimental trials (7.8%, *n* = 5).

### Adolescent mental health interventions in SSA

The 64 eligible studies describe a total of 57 unique adolescent mental health interventions across SSA. Supplementary Table 1 provides the detailed characteristics of these interventions. Three interventions were reported in more than one study; *Shamiri “thrive”* (26–28), *Suubi-Maka “hope for families”* (29–32)*, and Bridges to the future* (33–35). The common interventions were cognitive-behavioral therapy (CBT) (*n* = 20), family-based strengthening and economic empowerment interventions (*n* = 12), psychoeducation (*n* = 10, and interpersonal trauma-oriented narrative psychotherapy (*n* = 9).

### Mental health outcomes targeted by the identified interventions

The included studies reported a wide range of adolescents' mental health and psychosocial outcomes, with the most frequently reported outcomes being depression (*n* = 41), anxiety (*n* = 24), and post-traumatic stress disorder (PTSD) (*n* = 17) (Supplementary Table 1). Most of the studies (67.2%) focused on more than one mental health outcome. In addition to mental health outcomes, some studies assessed adolescent general health, as well as conduct outcomes such as violence, theft, bullying, and behavioral functioning. Three studies incorporated assessments of outcomes on parental or caregiver mental health and emotional functioning (23, 36, 37). Three studies also included outcomes on parental and adolescent bonding, connectedness and communication (23, 36, 38).

Although diverse tools were used to assess adolescent mental health outcomes, the Child Depression Inventory was the most commonly used measure of adolescent depression symptoms (29, 30, 33, 39–42) while the commonly used measures of PTSD included the University of California at Los Angeles Posttraumatic Stress Disorder Reaction Index (UCLA-PTSD) (41, 43–48) and the Posttraumatic Stress Diagnostic Scale (21, 49–52). The Revised Children's Manifest Anxiety Scale (RCMAS) and the Child and Adolescent Symptom Inventory (CASI) Anxiety Scale were the most frequently used measures of anxiety symptoms among adolescents (36, 42, 53–55).

Five studies assessed how adolescents' knowledge of HIV transmission impacted adolescent mental health (23, 36, 40, 56, 57). A total of fourteen studies included a measure that focused on either of the following adolescent outcomes: unresolved grief, (41, 58, 59) self-esteem, (22, 26, 40, 60) hopelessness, (32–35, 61, 62) self-concept, (23, 32, 33, 35, 63) resilience (58, 60) and self-efficacy among adolescents (22, 37, 40).

### Intervention duration and providers

There was considerable variation in the duration of the interventions, the number of sessions, and content across the included studies. Overall, the interventions lasted between 1 week to 5 years with a median duration of 3 months (Inter Quartile Range: seven weeks to 12 months). Five of the interventions lasted more than 2 years in which four (33, 34, 42, 64) involved economic strengthening and financial support for families of the adolescents, while one study (45) entailed creative play and psychotherapy (33, 34, 42, 64).

Most interventions were implemented by lay providers while 18 of them (28.0%) were implemented by professional psychologists. The lay providers included individuals without prior psychology training such as teachers, (43, 63, 65) community facilitators (48, 52), and youth ambassadors trained on the intervention component and the implementation process by psychologists who were mostly the lead researchers. Five of the interventions were cash transfer programmes implemented by the government (42, 62, 66–68) while two studies did not report on the providers of the intervention (64, 69). Only one integrated intervention that targeted reducing depression and anxiety was conducted by both lay and professional practitioners in Malawi and Tanzania (70).

### Population targeted

The eligible studies recruited a total of 40,072 adolescents from diverse populations and varying sample sizes ranging from 11 to 5,514. Most of the interventions described in the included studies were selective interventions that targeted adolescents at high risk of developing mental health problems including HIV-affected adolescents (*n* = 16), war-affected adolescents (*n* = 12), orphans (*n* = 17), adolescents from a poor background (*n* = 4), unmarried teenage mothers (*n* = 2), children of migrants (*n* = 1) and survivors of sexual violence (*n* = 4). Ten of the included studies described universal interventions targeting the general population of adolescents (26, 27, 55, 62, 65, 67, 69–72). Only four of the included studies described interventions that targeted adolescents with diagnosed mental and developmental disorders including depression and anxiety, (27, 61), PTSD (73), and autism (53) (Supplementary Table 1).

### Mode of delivery of the interventions

The majority of the interventions (*n* = 44) were group interventions and entailed diverse activities implemented in schools and at the community level. They included group CBT, psychoeducation on trauma, economic strengthening, family strengthening programme, and skills acquisition programmes. Only seven of the interventions were exclusively delivered in a one-on-one environment and involved interventions tailored to the specific needs of the individual (Supplementary Table 1). One of the studies reported the use of individual-based trauma-focused narrative exposure therapy provided by local lay counselors in treating PTSD among traumatized former child soldiers in Uganda (50). The study reported that the intervention not only resulted in a significant reduction in PTSD symptoms, but also reduced depressive symptoms, and suicidal ideation among former child soldiers. Other interventions were delivered as a blend of one-on-one support and group activities, (21, 51, 56, 74) and were all associated with a significant reduction in mental health problems.

### Settings of delivery of the interventions

The identified interventions were implemented in diverse settings including schools, churches, camps, and health facilities, among others. Therefore, we classified the interventions into community-based interventions, school-based interventions, and facility-based interventions. Notably, some interventions were implemented in more than one setting (30, 33, 35, 38, 39, 48, 62, 64, 70, 75).

### Community-based interventions

Most of the identified interventions (*n* = 44) among the eligible studies were community-based mental health interventions and emanated from 13 countries (Supplementary Table 1). Half (*n* = 22) of these interventions were implemented in only three of the 13 countries (Kenya, South Africa, and Uganda). Two studies reported on multi-country interventions (49, 70). Seven studies reporting community-based interventions reported on interventions that solely targeted girls (26, 44, 51, 57, 60, 76), while also other seven interventions targeted adolescents who were victims of war (21, 38, 43, 51, 76–79) with one of them aimed at displaced girls who had survived insurgency (76), and one targeted former female child soldiers (51). Nine studies targeted orphans and vulnerable adolescents from poor backgrounds (30, 37, 41, 62), with one of the studies focusing on AIDS orphaned adolescents (30). Overall, four interventions were aimed at improving the mental health of HIV-affected adolescents (30, 32, 40, 80). Ten of the community-based interventions were home-based and involved family meetings involving caregivers, parents, and adolescents. Psychosocial therapies and components drawn from Trauma-focused CBT were identified in fifteen studies as prominent implementation strategies employed by the community interventions.

Other interventions were carried out in camps in Sierra Leonne (81), Botswana (58), and Tanzania (22), with the duration of the intervention ranging from 1 to 4 weeks. Camp-based interventions included the use of Memory Work Therapy, group therapy, art therapy, sports or game activities and music therapy, outdoor challenge, and team-building activities (22, 58, 81).

### School-based interventions

Schools were the second most common setting in which the interventions were implemented with 19 mental health interventions implemented exclusively in schools (Supplementary Table 1). The interventions targeted diverse groups of adolescents in school settings including all students (26, 65, 82, 83), orphans and vulnerable adolescents (54, 57, 84), and students at increased risk of developing mental health problems such as those living with HIV (29, 36, 63, 85), war-affected adolescents (81, 86), and those diagnosed with mental health disorders (53, 73). The most targeted mental health outcomes by school-based interventions were depressive symptoms, PTSD, and anxiety symptoms (Supplementary Table 1).

Seventeen studies provided information about intervention facilitators for school-based interventions, in which 11 interventions were delivered by trained lay providers (27–29, 35, 48, 52, 54, 57, 63, 65, 83), while six (24, 33, 53, 84, 87) were delivered by professionals (24, 33, 53, 73, 84, 87). Fourteen (24, 27–29, 33, 34, 48, 52–54, 63, 82–84, 87, 88) of the interventions were delivered in groups (24, 28, 29, 33, 48, 52–54, 63, 68, 82) while three (57, 65, 73) were delivered in one on one sessions. The common implementation strategies in school-based interventions were economic-based interventions entailing financial support such as payment of school fees (29, 30, 32–35, 38, 64, 67, 68), and CBT (38, 48, 52, 53, 69, 86).

### Facility-based interventions

Health facility/clinic-based interventions were the least common and were reported in three studies conducted in health care facilities in Uganda (39), Tanzania (47), and Nigeria (61). The *Suubi* + *Adherence* intervention was a multicomponent intervention that was implemented among adolescents living with HIV in Uganda (39). The intervention included sessions on setting short-term and long-term goals, financial management seminars, income-generating activity training, medical assistance, and psychosocial support. Lay counselors trained in ART counseling provided psychosocial support to patients in HIV clinics.

The *Sauti ya Vijana (SYV; The Voice of Youth)* intervention was aiming at addressing mental health challenges to improve HIV outcomes among adolescents admitted to two HIV clinics (47). The intervention entailed providing a safe space where participants could share their past experiences.

The Nigerian study assessed the effectiveness of CBT among adolescents with clinically diagnosed depressive disorder attending a specialist psychiatric hospital (61). The group format intervention was administered by a psychiatrist in four weekly sessions using interactive lectures and group discussions. The sessions entailed psychoeducation, promoting hope and medication adherence, and an activity schedule.

### Effectiveness of the interventions

Sixty-two studies examined the effectiveness of the interventions, with 55 of them finding a positive significant impact on various mental health outcomes. Firstly, for the community-based interventions, they included a variety of activities targeted at preventing HIV, strengthening family relationships, and reconciliation in addition to supporting mental health among adolescents. Most of the community interventions entailed psychosocial therapies with components derived from Trauma-focused CBT (22, 38, 41, 44, 46, 48, 49, 58, 79, 81, 89). All the studies that reported on CBT intervention reported that CBT significantly reduced mental health problems among adolescents and their family members (Supplementary Table 1) (22, 38, 41, 44, 49, 58, 81). The Family Strengthening Intervention (FSI), for example, significantly improved the mental health outcomes of the entire household and strengthened child-caregiver interactions, family communication, HIV psychoeducation, and resource provision (38). In addition, unconditional cash transfers intervention was associated with improved psychological wellbeing among adolescents (42, 62, 66).

Results of school-based interventions showed that most interventions (17/19) were effective in improving the mental health and wellbeing of students. According to Olowokore et al. (54), the Resilience Training intervention reduced depressive symptoms and improved social connection, and self-esteem among children. However, this intervention did not have any significant effects on anxiety among the adolescents.

The *Suubi* (Hope) Economic Strengthening Intervention, which was implemented in Uganda and included activities such as counseling and mentorship, the provision of scholastic materials, and matched savings accounts for students, resulted in improved physical health and a decrease in depression and hopelessness (30). In addition, another study conducted in Kenya reported that school support in the form of tuition payments and the provision of scholastic materials such as school uniforms may help to cushion against the onset and exacerbation of mental health problems (64).

In addition to improved mental health outcomes, school-based mental health interventions significantly improved other positive outcomes such as school enrollment, school attendance, and discipline (38, 62, 64, 66). Other reported positive outcomes of the school-based interventions included better emotional regulation, increased prosocial support, reduced functional impairment, and reduced health risk behaviors. However, one school-based intervention had no significant impact on mental health although it improved other aspects of school outcomes such as school attendance (38).

Three healthcare-based studies reported a positive impact of the interventions on improved mental health outcomes among adolescents (39, 47, 61). Post-intervention follow-up and evaluation of the family-based economic intervention indicated that the intervention was effective in enhancing the mental health of adolescents living with HIV (39). The one-week post-intervention evaluation of the Nigerian study also indicated a statistically significant reduction in depressive symptoms and also improvements in the adolescents' knowledge of depression, hope, and attitude toward treatment adherence.

## Discussion

This scoping review aimed to identify and describe interventions that promote mental wellbeing among adolescents in SSA. A total of 64 studies describing 57 interventions were identified. Of the identified interventions, more than half of them (53.1%) were from only three countries in SSA (Uganda, Kenya, and South Africa). This potentially reflects a low coverage and prioritization of mental health interventions across SSA which points to an urgent need for investment in adolescent mental health promotion in the region.

Our review revealed that the existing mental health interventions in SSA targeted a variety of adolescent subpopulations (for example, adolescents living with HIV, adolescents affected by war, and adolescents from low-income families) and were implemented in diverse settings, including schools, communities, and health facilities. This is important as it shows that mental health interventions can be delivered outside conventional healthcare settings. Moreover, it is encouraging to note that most of the interventions were delivered by lay workers. This is particularly important for SSA settings where there is limited mental health infrastructure, including mental health professionals. This noted, the diversity of adolescent sub-populations requiring mental health interventions and services points to a need for evidence-based mental health promotion as well as a need for contextually appropriate tailoring of mental interventions to enhance uptake and effectiveness.

In this review, we observe that CBT is the most frequently utilized mental health promotion intervention, which may be attributable to its effectiveness as a problem-oriented strategy based on the findings from this review and elsewhere (90). First, in all the studies that utilized CBT in this review, significant reductions in mental health problems among adolescents and that of their family members were reported (see Supplementary Table 1). Second, similar findings have been observed in a systematic review on the effectiveness of CBT interventions (91). Some of the common components found in successful CBT interventions are behavioral activation, goal setting, relaxation, affective modulation, cognitive coping, trauma narrative, stress management/relaxation techniques, creating a trauma narrative, and cognitive processing. However, we could not determine which components were the most effective because they were mixed and blended in the interventions.

On the other hand, digital-based interventions were the least utilized interventions. This is despite the availability of evidence on the use and effectiveness of technology in delivering mental health interventions in other settings (92). This might be a reflection of the state of technology in SSA compared to other regions (93). Some interventions involved household economic strengthening through socio-economic empowerment programmes, such as improving access to education, credit schemes, cash transfers, savings schemes, and financial management training. The findings of the study suggest the potential of economic empowerment interventions in decreasing psychological distress among vulnerable adolescent populations.

With regards to community-based interventions in SSA, results from the included studies are promising given the positive impact that was shown by the interventions across a diverse range of mental health outcomes. Most of the community-based interventions reported improvement in mental health outcomes of adolescents following the intervention. Mostly, community-based interventions were aiming at reducing psychosocial issues by fostering parent-child relationships, improved-caregiver relationships, family communication, and parenting skills, connections to resources, and HIV psychoeducation. A key implementation strategy for community-based interventions was the use of community-based organizations and the involvement of local community members, including community health volunteers, chiefs, and religious leaders in the delivery of the intervention. One component that contributes to the success of these treatments is the collective mobilization of resources and sensitization of communities, which enhances responsiveness to adolescent needs and breaks down barriers to obtaining mental health services.

For the family-focused interventions, study implementers, including psychologists and counselors, were able to reach as many vulnerable adolescents as possible in the comfort of their homes, thus reducing barriers, such as transportation, that many poverty-stricken families face when trying to access health facility or center-based mental health interventions. Although the results of family-focused interventions are promising, future research should investigate the sustainability of such interventions.

Camp-based recreational and creative activities such as outdoor challenges, sports, and games had a positive effect not only on mental health outcomes but also on resilience, social-emotional changes, behavioral outcomes, and physical activity among adolescents. It is encouraging to note that all the camp-based interventions reported that the interventions were associated with an increase in resilience and self-esteem.

With regards to the school-based interventions, the findings demonstrate that school-based programs implemented across SSA can have a significant positive impact on adolescent wellbeing, including reduced PTSD, depression, and anxiety, as well as enhanced coping strategies. Most of the school-based interventions were delivered by trained teachers (26, 28, 29, 35, 52, 54, 57, 63, 65), with the remaining interventions implemented by professionals, particularly psychiatrists and psychologists (33, 53, 73, 86). This suggests that trained teachers can effectively deliver mental health promotion interventions. The use of teachers to implement school-based interventions may be a long-term and low-cost strategy for improving mental health outcomes in children since they may continue making use of some of the interventions' components even after the research project is completed.

Another group of interventions targeted adolescents in health care facilities, particularly in HIV clinics and psychiatric hospitals. One of the interventions included psychoeducation and incorporated components of CBT on adolescents with clinically diagnosed depressive disorders under treatment with antidepressants (61). It is difficult to determine the more effective treatment of mental health problems between the use of medication and psychosocial interventions. Studies that combined the two treatment options yielded promising results, implying that combining psychosocial interventions such as psychoeducation and CBT with antidepressant medication is viable, acceptable, and can provide a more positive impact on depressed youths in the region.

## Strengths and limitations

This review's main strength was its rigorous and comprehensive nature in which we searched five major databases, including an African-based database, as well as a search of gray literature. Another strength of the review lies in its ability to explore a timely research theme in a geographical context where evidence synthesis on this subject is still limited yet critical for informing interventions.

This review also has limitations worth highlighting. Although most of the studies used randomized study designs, differences in terms of study settings, types of interventions, length of interventions, sample size, and measures made direct comparisons and pooling of effects of the interventions challenging, thus a narrative synthesis was undertaken.

Lastly, another limitation of the current review worth noting is a challenge common to scoping reviews. The quality and the risk of bias of the included studies were not assessed and therefore, the level of certainty on the effectiveness of the interventions is limited. Lastly, the adolescent population is a heterogeneous age group (e.g. early and late adolescence) which may experience the effects of the interventions differently. However, we were unable to disaggregate our findings by the adolescent age sub-categories.

## Future research

Overall, in regards to informing future research, this scoping review informs mental health programmers of the variety of mental health interventions among which suitable choices can be adapted to settings and developmental stages to optimize effectiveness. The scoping review points to a need for developmentally appropriate interventions considering that the adolescent period includes both early and late adolescence, whose mental health needs and burdens may differ and thus require a targeted approach.

## Conclusions

We identified 57 unique adolescent mental health interventions across SSA. These interventions can be administered to diverse sub-populations and can be implemented in a variety of contexts, including schools, communities, camps, and clinical settings. They can also be administered by different providers, including lay workers, which is particularly important for SSA. Most of the interventions were reported to be effective against the targeted mental health outcomes. However, it is difficult to ascertain the strength of this evidence as we did not assess the quality of the included studies.

## Data availability statement

The original contributions presented in the study are included in the article/Supplementary material, further inquiries can be directed to the corresponding authors.

## Author contributions

AA conceptualized the study. AA, AM, ET, GM, and EC refined the search terms. GM and AM did the database search. GM and AM screened the articles and did the data extraction of included studies, while AA provided guidance throughout the process. The first draft of this article was written by AM, while GM, EC, ET, AS, MN, JA, DS, and AA critically reviewed and contributed to all the subsequent manuscript revisions. All the authors except the late Adam Mabrouk read, reviewed, and approved the final manuscript.

## Funding

This publication was produced with the financial support of the European Union (Contract No: CSO-LA - 2020 / 418 - 235). Its contents are the sole responsibility of the authors and do not necessarily reflect the views of the European Union. The funders had no role in the study's design, in the collection, analyses, or interpretation of data, in the writing of the manuscript, or in the decision to publish the results.

## Conflict of interest

The authors declare that the research was conducted in the absence of any commercial or financial relationships that could be construed as a potential conflict of interest.

## Publisher's note

All claims expressed in this article are solely those of the authors and do not necessarily represent those of their affiliated organizations, or those of the publisher, the editors and the reviewers. Any product that may be evaluated in this article, or claim that may be made by its manufacturer, is not guaranteed or endorsed by the publisher.
