# Supplementary material for: Mental health interventions for adolescents in sub-Saharan Africa: A scoping review
Source: Front Psychiatry. 2022 Aug 11;13:937723. doi: 10.3389/fpsyt.2022.937723 (PMC9429610; doi:10.3389/fpsyt.2022.937723)
Supplement: Supplementary file 1 [file Table_1.pdf]

Supplementary Table 1: Summary of the included studies

**Author, Year of publication, Country**	**Study design**	**Intervention setting**	**Participants, Sample size(n)**	**Age in years (range or mean or)**	**Type of intervention**	**Intervention name (If applicable) & components**	**Target mental health outcome/ Assessment tool used**	**Findings**
Angeles et al, 2019, Malawi	RCT	Community	Adolescent form poor households (n=2782)	13-19	UCT	***Malawi Social Cash Transfer Program*** Unconditional cash transfer of approximately 17-23% of median household consumption transferred bimonthly. Although the transfer was unconditional, there was messaging to urge beneficiaries to use it to invest in children's human capital and to meet basic household necessities.	Depressive symptoms- *Center for Epidemiologic Studies Depression Scale (CES-D)*	The program reduced indications of depression by about 15 percentage points (p<0.005)
Bell et al, 2008, South Africa	RCT	Community	HIV affected adolescents (n=5514)	9-13	Psycho-education	***CHAMPSA- Collaborative HIV Adolescent Mental Health Pro-gram South Africa*** Use of a cartoon-based story line of a 12-year-old boy orphaned by AIDs, comprises 10-90 minute sessions designed to increase HIV knowledge and decrease stigma surrounding HIV infections, increase connectedness to caregiver social networks	General health- *The General Health Questionnaire* Anxiety- *Revised Children's Man-ifest Anxiety Scale* AIDS transmission knowledge- *AIDS Transmission Knowledge Scale* Stigma- *Stigma Scale*	No significant impact on mental health (P*< 0.3527, ES=1.00 in the TG vs, ES= 0.23 in the CG) Significant positive effect on HIV transmission knowledge (P*<0.0084, ES=0.631), and less stigma toward HIV-infected people (P*<0.0187, ES=0.403)
Betancourt et al, 2014, Sierra Leone	RCT	Community & schools	War affected adolescents (n=436)	18.0 (SD:2.4)	CBT and IPT	***Youth Readiness Intervention (YRI)*** Psychoeducation about trauma and its impact on interpersonal relationships; self-regulation and relaxation skills (i.e., deep abdominal breathing); cognitive restructuring (i.e., addressing negative self-perceptions due to trauma); behavioural activation; communication and interpersonal skills; and sequential problem solving. After YRI Participants randomly assigned to receive free education dubbed Educ-Aid	PTSD- *University of California, Los Angeles (UCLA) Post-Traumatic Stress Disorder Reaction Index (PTSDR)* Psychological distress- *Oxford Measure of Psychosocial Adjustment*	The treatment and control conditions did not differ significantly on improvement in psychological distress (β = -0.021, CI= -0.196 to 0.154, δ = -0.03) or the exploratory outcome of posttraumatic stress symptoms (β = -0.007, CI= -0.097 to 0.083, δ = -0.02)
Betancourt et al, 2015, Rwanda	Open trial	Community	HIV affected adolescents (n=39)	12.7 (SD:3.4)	Family-based preventive intervention (FBPI)	***The Family Strengthening Intervention (FSI)*** The FSI had the following components: Building parenting skills and improving family communication; developing a family narrative to increase family connectedness and hope, and highlight family resilience in the face of adversity; providing psychoeducation on HIV; and strengthening problem-solving skills and social support	Depression- *Center for Epidemiological Studies Depression Scale for Children (CESDC)* Anxiety/depression- *Youth Self-Report*	The number of children in the clinical range for depression dropped from five of 32 (15.63%) at baseline to four of 31 (12.90%) at post assessment and three of 33 (9.09%) at follow-up.

Bhana et al, 2014, South Africa	RCT	Community	Adolescent living with HIV (n=65)	10-13	Family strengthening intervention	***The VUKA Family Program*** A three-month, ten-session intervention that included the use of a cartoon-based story line about a 12-year-old kid orphaned by AIDS. Components included: (1) AIDS-related loss and bereavement; (2) HIV transmission and treatment knowledge; (3) Disclosure of HIV status to others; (4) Youth identity, acceptance and coping with HIV; (5) Adherence to medical treatment; (6) Stigma and discrimination; (7) Caregiver-child communication, particularly on sensitive topics such as puberty and HIV; (8) Puberty	Youth mental health- *Strengths and Difficulties Questionnaire* Depression- *Child Depression Inventory*	Observed reduction in depressive symptoms, mean score of 3.31 at baseline VS 2.03 at follow up in the intervention arm. While mean scores of control was 3.19 at baseline but after follow- up it was 2.64
Bolton et al, 2007, Uganda	RCT	Community	Survivors of War (n=314)	14-17	Interpersonal Psychotherapy	***IPT-G & CP*** Interventions comprised of 16 weekly group meetings, lasting about 2 hours. IPT-G focused on identifying the interpersonal problems linked to depression and then assisting the individual to manage the problems	Depression- *local depression symptom scale*	Difference in change in adjusted mean score for depression symptoms between group IPT and control groups was 9.79 points (95% CI: 1.66-17.93). Change in mean score was higher among Girls receiving IPT (12.61 points; 95% CI, 2.09-23.14) compared to controls No significant Improvement among boys Creative play showed no effect on depression severity
Cavazos-Rehg et al, 2021, Uganda	RCT	Community/ Clinics	Adolescents living with HIV (n=702)	10-16	Family-Based Economic Intervention	***Suubi + Adherence*** Included four sessions on financial management and training in income-generating activities and combined a matched savings account—opened in the child and caregiver names.	Hopelessness- *The Beck Hopelessness scale* Depressive symptoms- *Children's Depression Inventory (CDI)* Self-concept- *Tennessee Self-Concept Scale*	The intervention arm had a statistically significant lower score on depression than the control group at 36-month follow-up (4.94 vs. 5.81; p = .029)
Dorsey et al,2020 Kenya and Tanzania	RCT	Community	General population of adolescents (n=640)	10.6 (SD:1.6)	Trauma focused CBT	*Pamoja Tunaweza: "Together We Can"* Entailed psychoeducation and coping strategies, behaviour management skills. TF CBT entailed 12 group sessions and 3 to 4 individual sessions for 12 consecutive weeks	PTSD- *Child PTSD Symptom Scale*	Trauma-focused CBT was more effective than usual care (UC) for PTS in 3 of 4 sites after treatment (after end of 3-month RCT): rural Kenya (Cohen d = 1.04 [95% CI: 0.72-1.36]), urban Kenya (Cohen d = 0.45 [95% CI: 0.10-0.80])

						Child groups were led by 2 counsellors, while guardian group sessions were led by 1 counsellor		At t 12 month follow-up, TF-CBT was more effective in reducing PTS only among children in rural and urban Kenya
Ertl et al, 2011, Uganda	RCT	Community	Former child soldiers (n=85)	18.0 (SD:2.0)	Trauma-focused therapy (narrative exposure therapy)	Intervention entailed a narrative exposure therapy of 8 individual sessions by trained local lay therapists, academic catch-up with counselling. Sessions lasted between 1 hour to 2 hours and were scheduled 3 times a week.	PTSD- *Clinician-Administered PTSD Scale (CAPS)* Depression- *Mini International Neuropsychiatric Interview (MINI)* Suicide risk- *module C of the MINI*	Significant reduction in PTSD symptom in the narrative exposure therapy group than in the academic catch-up (mean change difference, −14.06 [95% confidence interval, −27.19 to −0.92]) and waiting-list (mean change difference, −13.04 [95% confidence interval, −26.79 to 0.72]) groups. There was also significant reduction in depressive symptoms and suicidal ideation,
Green et al, 2019, Kenya	RCT	Schools/ Community	Orphaned adolescents (n=835)	14.8 (SD:1.5)	Scholastic support	***School support intervention*** Payment of school tuition fees for secondary school; provision of a school uniform in primary school and the first year of secondary school; and nurse visits	Depressive symptoms- *Center for Epidemiologic Studies Depression Scale Revised (CESD-R)*	School support intervention prevented depression severity scores from increasing overtime among adolescents recruited from intervention schools (effect size of 0.28)
Han et al, 2013, Uganda	RCT	Schools	Children living with HIV-AIDs (n=297)	13.4 (1.3)	Family economic empowerment	***Suubi-Maka: "hope for families"*** The intervention entailed, providing adult mentors to children, financial management workshops, and promoting monetary savings for educational opportunities	Hopelessness- *Beck Hopeless Scale (BHS)* Depression- *Child Depression Inventory (CDI)*	Depression scores in the intervention arm reduced by almost 5 points (from 13.06 to 8.42) and a 2.66 reduction (from 13.24 to 10.58) in the control arm
Harding, 2019, Tanzania	RCT	Community	Orphaned children living with HIV (n=48)	14-18	Trauma focused narrative therapy	***Memory Work Therapy*** The aim was to create a safe space for the participants to explore their life story in an individual or group setting in five-day residential camp, and included Memory box, Memory book, Tree of life and the Hero (Active Citizen) book	Psychological problems- *The Brief Symptom Inventory (BSI)* Self-worth- *The Rosenberg Self Esteem Scale (SES)* Emotional and behavioural Affective disorders- *The Strengths and Difficulties Questionnaire (SDQ)*	Intervention effects were found on all total measures: Brief Symptom Inventory d = 0.99, p < .001; Rosenberg Self Esteem Scale d = 0.65, p = .001; Strengths and Difficulties Questionnaire d = 0.64, p = .002; Self-efficacy questionnaire for children both "social" and "emotional" subscales, d = 0.54, p = .008 and d = 0.63, p = .002, respectively
Ireri et al, 2019, Kenya	RCT	School	Children and adolescents ASD (n=40)	5-21	CBT	***The Multimodal Anxiety and Social Skills Intervention (MASSI)*** MASSI was grounded in CBT and had 13 sessions individual therapy each at least 60 min, 7 group therapy sessions (social skills training and practice) and family/school involvement.	Anxiety- *Child and Adolescent Symptom Inventory-4 ASD Anxiety Scale (CASI-Anx)* ASD severity- *parent-reported Social*	There was a 23% decrease in anxiety, in the treatment group. Interaction between time and condition was significant, F (2,78)=5.614, p=0.006, η2=0.067 for anxiety. Children in the treatment condition showed sharper decline in anxiety than did the control participants.

							Responsiveness Scale-second edition (SRS-2)	
Isa et al, 2018, Nigeria	RCT	Clinic	Adolescent diagnosed with depressive disorder (n=18)	13-18	CBT	The intervention was delivered weekly by a psychiatrist. Each group had around 10 participants and the session lasted for 40 minutes. Component entailed; Psychoeducation, promoting hope and medication, activity scheduling. Sessions delivered by a psychiatrist in 4 weekly sessions in a group format using interactive discussions and lectures.	Depression- *The Beck Depression Inventory (BDI)* Moods- *Short Mood and Feelings Questionnaire* Hope- *Children's Hope Scale*	Statistically significant reductions in depressive symptoms were recorded with an observed reduction in the mean BDI compared with baseline (3.94 (SD, 2.10) vs 24.4 (SD 11.18), p = 0.001)
Ismayilova et al, 2018, Burkina Faso	RCT	Community	Adolescents and children living in extreme poverty (n=360)	10-15	Economic strengthening intervention and family coaching component	***The Village Savings and Loan Association (VSLA) model*** Intervention entailed livelihood training and planning, savings group formation and training, one-to-one monthly mentoring, financial support to participants, family coaching for household members and seed capital grants.	Depression- *Center for Epidemiological Studies Depression Scale for Children/CES-DC)* Self-esteem- *Rosenberg Self-Esteem Scale/RSES* Trauma symptoms- *The Children's Revised Impact of Events Scale/CRIES-8*	Children who received the economic strengthening intervention plus the family coaching component (TU + group) showed a reduction in depressive symptoms at 12 months (medium effect size Cohen's d=−0.41, p=.001) and 24 months (d=−0.39, p=.025) compared to the control and the economic intervention alone (TU) (at 12 months d=−0.22, p = .020).
Jani et al, 2016, Ethiopia	Quasi	Community	Migrant children (n=730)	15-18	Psychosocial counselling intervention	Involved counselling components, including individual, group and creative therapies, such as music, art and drama. Counsellors used a client-centred approach, to address main issues brought by the client, provide possible options, and plan of action selected by the client.	Mental health problems- *The Youth Self-Report (YSR)*	For females, aggressive behaviour decreased by 60% (adjusted odds ratio (AOR): 0.4 (0.25 to 0.65)) and any mental health problem decreased by 50% (AOR: 0.5 (0.36 to 0.81)) from baseline to end line
Jordans et al, 2011, Burundi	Quasi-quantitative-qualitative analysis design	Community	Children in areas of armed conflicts with elevated psychological distress (n=11)	11-14	Nonspecialized Psychosocial counselling intervention	The intervention consisted of (a) a face-to-face engagement between a trained counsellornand a client; (b) a supportive process that was problem-specific and goal-directed; and (c) working toward reduction of a client's psychosocial problems.	Depression- *Depression Self-Rating Scale* Anxiety- *Child Anxiety Related Emotional Disorder* Posttraumatic symptoms (PTSD)- *Child Post Traumatic*	The study does not report on effectiveness of the intervention. However, five treatment themes appeared associated with outcome trajectories: client centeredness, therapeutic alliance, active problem solving, trauma- focused exposure, and family involvement. Higher levels

							Stress Disorder (PTSD) Symptom Scale	appeared associated with better outcomes<br>From the findings of the study, integrative counselling, which combines universal therapist variables (therapeutic alliance and client-centeredness) with active use of problem-focused therapeutic approaches (problem-solving and trauma-focused exposure) and a systemic perspective, may be an effective strategy for treating mental health symptoms in children in Burundi.
Karimili et al, 2019, Uganda	RCT	Community/ schools	AIDS orphaned children (n=1410)	10-16	A family-based combination intervention	***Bridges to the Future***<br>Economic strengthening and asset accumulation with savings. Workshops on family financial management and microenterprises development, nine session mentorship programme and a matched savings account	Child mental health- *Beck Hopelessness Scale, Child Depression Inventory, and Tennessee Self-Concept Scale*	Significant impact of the intervention on children's mental health at 24 months ((B=−0.59; 95% CI: 0.93, −0.25; p < 0.001; β=−0.33)
Katisi et al, 2019, Botswana	Quasi	Camp	Adolescents orphaned by HIV (n=650)	11-17	Trauma-focused therapy	***Balekane EARTH program***<br>Involved weeks of outdoor challenge and team building. 3 year planned periodic kinship peer group meetings, team building activities, home intervention by social workers for special cases that need immediate family reconciliation	Resilience- *Child and Youth Resilience Measure*<br>Impact of grief- *Inventory of Complicated Grief*	Over the course of the camp, overall resilience<br>scores significantly improved for both male (p = 0.001) and female participants (p = 0.014),<br>though effect sizes were small (r = 0.14 and = 0.10, respectively), and only 8% of females<br>and 11% of males demonstrated reliable change
Kilburn et al, 2016, Kenya	Cohort	Community	Orphans and vulnerable children from poor families (n=1960)	11-20	CT-OVC	***Kenya's Cash Transfer for Orphans and Vulnerable Children program***<br>The program entails providing approximately US$20 to households that are poor and have at least one orphan or vulnerable child below 18 years of age.	Depression- *CES-D 10 scale*	The cash transfer reduced the odds of depressive symptoms by 24 percent among young persons living in households that received cash transfers (odds ratio [OR] 0.79;95% CI:0.63-0.99)
Kumakech et al, 2009, Uganda	RCT	Schools	HIV affected adolescents (n=326)	10-15	Peer-group support intervention	16 psychosocial exercises, which were implemented by school teachers over 10 weeks' period. Designed in a form a play or a game. Sharing of fears, worries and concerns about orphan hood, problem-solving; HIV/AIDS; fears about orphan hood and how to handle fear,	Depression and anxiety- *Beck Youth Inventories (BYI)*	The intervention had a significant impact on anxiety (p= 0.003), depression (p<0.001), and anger among AIDS orphans (p<0.001). Follow-up symptoms of anxiety, depression, and anger were lower than among the control orphans.
Kutcher et a, 2019, Malawi Tanzania	RCT	Community/ Schools	School going high school students (n=4000)	10-15	Integrated Approach to Addressing the Challenge of Depression	***An Integrated Approach to Addressing the Challenge of Depression Among the Youth in Malawi and Tanzania (IACD)***<br>Interactive radio programs and other electronic interventions, such as Facebook and WhatsApp, teacher training	Depression- *Assessment tool not reported*	Significant improvements in young people who listened to the program than those who had no exposure to it. No OR and effect sizes reported.

Leerlooijer et al, 2014, Uganda	Intervention mapping	Community	Unmarried teenage mothers (n=1036)	15-19	Incentives based intervention	***Teenage Mothers Project*** The project aims to improve psychological wellbeing of unmarried teenage mothers through, teenage mother support groups, formal education and income generation support, counselling, and advocacy	Psychological (Loneliness, self-esteem, feelings of isolation and lack of social support) and social well-being- *Assessment tools not reported*	The paper does not report on the impact the intervention had on psychological outcomes
Gupta et al, 2008 Sierra Leone	Cohort	Camp	War affected adolescents (n=315)	10.7	Trauma focused therapy	***Rapid-Ed literacy and numeracy*** A total of eight 1-hour trauma healing and recreation activities were implemented in camp classes twice per week for over a period of one month. Techniques utilised aimed to reduce children levels of both emotional and post-traumatic stress.	PTSD- *Impact of Events Scale (IES)*	The mean total IES score at the post-test was significantly lower than the mean total pre-test IES score (32.9 and 42.5respectively; t= 18.82, P < 0 .0001).
Mc Bain et al, 2015, Sierra Leone	RCT	Schools	War affected adolescents (n=436)	18.0 (SD: 2.4)	CBT	***Youth Readiness Intervention*** Psychoeducation about trauma, self-regulation and relaxation skills, cognitive restructuring, behavioural activation, communication and interpersonal skills and sequential problem solving	Depression and anxiety- *Oxford Measure of Psychosocial Adjustment*	Adaptive behaviour among YRI recipients was significantly higher than among controls (β=0.149, 95% CI 0.057–0.240), as were emotion regulation skills (β=0.109, 95% CI 0.026–0.191). In contrast, those in treatment and control conditions reported similar levels of internalizing (β=0.005, 95% CI: 0.089 to 0.100) and externalizing (β=0.032, 95% CI: 0.134 to 0.070) at post-assessment. At 6-month follow-up (6 months after post-assessment), those who received treatment again reported higher emotion regulation (β=0.269, 95% CI: 0.073–0.464) as well as lower externalizing (β=0.277, 95% CI: 0.527 to 0.027) and marginally greater levels of functioning (β=5.613, 95% CI: 0.131 to 11.357)

McMullen et al, 2013, Dr Congo	RCT	Community	Former child soldiers and war affected adolescents (n=50)	13-17	CBT	***TF-CBT***<br>15 group therapy sessions including; psychoeducation, stress management/relaxation techniques, affect expression and modulation, cognitive coping, creating a trauma narrative, cognitive processing, future hopes	PTSD- *UCLA-PTSD Reaction Index*<br>Psychosocial distress- *The African Youth Psychosocial Assessment*	TF-CBT group had highly significant reductions in posttraumatic stress symptoms [F(1, 45) = 89.27,p < .001, np ² = .665], overall psychosocial distress [F(1, 45) = 72.47, p< .001, np ² = .617], depression/anxiety-like symptoms [F(1, 45) = 58.82,p < .001, np ² 2 = .567], conduct problems [F(1, 45) = 18.18,p < .001, np ² = .288] and an increase in prosocial behaviour [F(1, 45) = 34.18, p< .001, np ² = .432]
Mueller et al, 2011, South Africa	Quasi	Community	HIV affected adolescents (n=297)	12.2	Arts and education activities	***MAD ('Make A Difference')***<br>Art and education activities to build a sense of self-worth (self-esteem), self-concept, empowerment and emotional control (self-efficacy).	Depression- *Child Depression Inventory (CDI)*<br>Emotional behavioural problems- *Strengths and Difficulties Questionnaire (SDQ)*<br>Self-esteem- *Rosenberg Self-Esteem Scale*<br>Self-efficacy- *The Self-Efficacy Questionnaire*	Those in the intervention arm showed significantly higher self-efficacy scores with an effect size of *B* = 3.61 (*P* < 0.05)<br>No impact of the intervention on depression *B* = -0.16 (*P* = 0.67), emotional and behavioural problems *B* = 0.23 (*P* = 0.75), and self-esteem *B* = 0.18 (*P* = 0.75)
Musuva et al, 2017, Kenya	RCT	Schools	School going adolescents (n=36)	9-12	*Praziquantel* mass-drug administration	The study assessed changes in behaviour after mass administration of praziquantel for *Schistosoma mansoni*	(Externalizing and Internalizing problems)- *Behavioural Assessment System for Children (BASC-2)*	BASC-2 T scores were significantly reduced post-treatment (p < 0.05) for both externalizing problems (hyperactivity, aggression, and conduct problems)- *t* = 2.97 and internalizing problems (anxiety, depression, somatization, a typicality, and withdrawal)- *t* = 2.59
Newnham et al, 2015, Sierra Leone	Feasibility study, no control arm	Community	War affected youth (n=32)	18.2 (SD:2.4)	CBT	***The Youth Readiness Intervention***<br>Interventions included closed group sessions that entailed relaxation techniques, assertive communication strategies, cognitive restructuring, behavioural activation, goal setting, and sequential problem solving	(Internalizing and Externalizing symptoms)- *The OMPA*<br>Functional adaptation- *WHOQOL-BREF*<br>Emotional regulation- *Difficulties in Emotion Regulation Scale*	Significant reduction in externalizing (Cohen's *d* = 0.79), internalizing symptoms (*d* = 0.88), significant improvements in adaptive behaviour(*d* = 0.49) , and emotion regulation (*d* = 0.74)

O'Callaghan et al, 2013, DRC	RCT	Community	Sexually exploited girls (n=52)	12-17	Trauma focused CBT	Intervention entailed 15 sessions that included the following modules: introduction (ground rules, psycho-education on rape and trauma, and a safe place); stress management (controlled breathing, progressive muscle relaxation, and thought stopping); feelings (affect expression and modulation); cognitive coping (the cognitive triangle, the relationship between thoughts, feelings, and behaviour; trauma narratives; and identifying and changing inaccurate or unhelpful cognitions.	PTSD- *UCLA PTSD Reaction Index* Psychosocial functioning (Depression, anxiety, conduct behaviours, and prosocial behaviours)- *The African Youth Psychosocial Assessment Instrument (AYPA)*	Intervention group experienced significant reductions in PTSD symptoms compared to control ($F_{1,49}$ = 52.708, p < 0.01, x^2p = 0.518), a reduction in depression and anxiety ($F_{1,49}$ = 52.371, p < 0.01, x^2p = 0.517), a reduction in conduct problems ($F_{1,49}$ = 17.123, p < 0.01, x^2p = 0.259), and a significant increase in prosocial behaviour ($F_{1,49}$ = 5.39, p < 0.05, x^2p = 0.099)
O'Donnell et al, 2014, Tanzania	Open trial	Community	Orphaned children (n=64)	10.8 (SD:1)	Trauma focused CBT	Components covered in the CBT: Psychoeducation, parenting, relaxation, affective modulation, cognitive coping, trauma narrative and processing, in vivo exposure, and enhancing safety	Unresolved grief- *Grief Screening Scale (GSS)* PTSD- *UCLA Posttraumatic Stress Disorder-Reaction Index* Depression- *The Short Mood and Feelings Questionnaire (SMFQ)* Behavioural emotional wellbeing- *Strengths and Difficulties Questionnaire (SDQ)*	Reduced symptoms on PTSD (*B* = -20.78, *SE* = 19.1),unresolved grief (*B* = -6.69, *SE* = 0.75) depression (*B* = -7.84, *SE* = 0.87), and behavioural wellbeing (*B* = -6.69, *SE* = 0.75) 12 months after intervention
Olowokere et al, 2014, Nigeria	Quasi experiment	Schools	Vulnerable children based on a verifiable criteria (n=109)	12.8 (SD:1.6)	Resilience training module on basic life skills	Peer support group and weekly lecture package of 6 session comprising of the following core resilience components: self-resilience, being oneself, having a sense of purpose, maintaining balance and harmony with self, having a sense of purpose, and perseverance	Anxiety- *18- scale tool developed by the authors* Depression- *Center for Epidemiological Studies Depression Scale for Children* Self-esteem- *Rosenberg self-esteem scale* Social connectedness- *Social Connectedness scale*	No significant difference in anxiety scores between the intervention and control arm (*M* = 5.37, *t* = 0.870, *p* = 0.386) Compared to the control group, the intervention arm observed a significant reduction in depression score (*M* = -4.94, *t* = -2.26, *p* = 0.03), increase in self-esteem scorers(*M* = 3.27, *t* = -0.2.26, *p* = 0.03) and a significant increase in social connection (*M* = 2.86, *t* = 3.16, *p* = 0.002)
Osborn et al, 2020, Kenya	RCT	Schools	High school students (n=103)	13-18	Computerized single-session interventions (SSIs)	***Shamiri-Digital (Shamiri means "thrive" in Kiswahili)*** Intervention entailed three modules: growth mind-set, gratitude, and value affirmation. Participants learned about responding to challenges, they also wrote their own growth stories about a	Depression- *Patient Health Questionnaire* Anxiety- *Generalized Anxiety Disorder Screener* Adolescent well-being- *Warwick*	A greater reduction in depressive symptoms in the intervention arm compared to the control arm (*p* = 0.028, d = 0.50 [.00, 1.6]) at 4 weeks' post-intervention No significant effects on anxiety symptoms (*p* = 0.280, *d* = .29 [-.20, .79]

						challenge they faced, and also learned about the importance of practicing gratitude	*Edinburgh Mental Well-being Scale*	
Osborn et al, 2020, Kenya	RCT	Community	Adolescents with depressive and/or anxiety symptoms (n=51)	12-19	Group Intervention that incorporated CBT and positive psychology	***The Shamiri and study skills***<br>Group based sessions that included reading activities, group discussions, and writing activities; 1st session: students wrote their own personal growth stories; 2nd: Students identified a challenge, brainstormed and discussed effective strategies/solutions; 3rd: Learned about gratitude; 4th: Students learned about values	Anxiety- *Generalized Anxiety Disorder Screener* Depression- *Patient Health Questionnaire*	Intervention resulted to greater reductions in adolescent depression symptoms (p = 0.038; d = .32) and anxiety symptoms (p = 0.039; d = .54)
Osborn et al, 2021, Kenya	RCT	Schools	High school students (n=413)	13-18	Layperson-delivered group intervention	***Shamiri intervention***<br>School based group based intervention consisting of 4 sessions made up of 3 modules: growth mind-set (two sessions), gratitude (one session), and virtues (one session)	Anxiety- *Generalized Anxiety Disorder Screnner* Depressive symptoms- *Patient Health Questionnaire*	Both the intervention (*Shamiri*) and the control (life skills) were rated highly useful in reducing both depressive and anxiety symptoms (4.8/5.0)<br>Youths in the intervention arm (*Shamiri*) showed greater reductions in both depressive symptoms (Cohen d = 0.45 [95% CI, 0.19-0.71]), and anxiety symptoms (d = 0.44 [95% CI, 0.18-0.71]) at 7 months' post treatment than the control group who received study skills
Puffer et al, 2016, Kenya	Stepped wedge cluster RCT	Community	General populations of adolescents (n=237)	12.3 (SD:2.0)	Family relationships strengthening intervention	***READY***<br>Intervention was delivered in 2-hours sessions totalling 9-session. The sessions were divided into 3 modules: economic empowerment, behavioural parent training, and CBT. The intervention in cooperated strategies from family communication skills training, economics, skills-based HIV prevention interventions, behavioural parent training, and cognitive behavioural therapies	Anxiety- *Multi-Dimensional Anxiety Scale* Depression- *Children's Depression Inventory* Self-esteem- *Rosenberg Self-Esteem Scale*	No measurable effect of the intervention on youth mental health outcomes
Richards et al, 2014, Uganda	RCT	Community	General populations of adolescents (n=1462)	11-14	Sports for development intervention	***Gum Marom Kids League (GMKL)***<br>The intervention was delivered as an 11-week voluntary competitive sport-for development football league. Each weekend the participants took part in a 40 minute game of football (boys: 11-a side full field; girls: 7-a-side half field) and various peace-building activities	Mental health status (Depression & anxiety)- *Acholi Psychosocial Assessment Instrument (APAI)*PTSD)- *Assessed by items from Acholi Psychosocial Assessment Instrument*	At 4 months follow, there was a negative effect on depression symptoms (ES = 0.67 [0.33 to 1.00]) and anxiety like symptoms (ES = 0.63 [0.30 to 0.96]) when comparing boys in the intervention group *vs* waitlist<br>No significant effect on the girls for any outcome

Robjant et al, 2019, DRC	RCT	Community	Former female child soldiers (n=92)	18 (16-25)	Narrative exposure therapy	***Forensic Offender Rehabilitation (FORTNET)***<br>Intervention was delivered in 6 individual sessions of 90-120 minute which took place every week initiated locally trained therapists. Sessions were about tapping and exploring traumatic events and positive moments and relationships; psychoeducation and basic skills on emotion regulation. In addition, each woman had one group therapy session per week lasting between 60 and 90 min.	PTSD- *PTSD Symptom Scale*<br>Depression- *Patient Health Questionnaire*<br>Aggression- *Appetitive Aggression Scale*	There was clinically significant improvement for PTSD symptom severity in 85% of women in the intervention arm compared to 46% in the control group (treatment as usual (TAU)), and improvement of 54% of cases for depression in the intervention arm compared to 39% in the control arm.
Rossouw et al, 2018, South Africa	RCT	Schools	Adolescent with PTSD (n=63)	13-18	Prolonged Exposure Therapy for Adolescents (PE-A)	***PE-A intervention and supportive counselling***<br>The intervention consisted of a 7-14 weekly, 1- hour client-centred therapy. The sessions focused on establishing a trusting and an empowering therapeutic relationship. The session was patient centred. Were participants directed the sessions, and choose what, and how to address the trauma. Participants were provided with active listening, empathy and encouragement to talk about their feelings.	PTSD- *Mini International Neuropsychiatric Interview for Children and Adolescents*<br>Functional impairment- *The Children's Global Assessment Scale*<br>Depression- *BDI*	Participants in the intervention arm, experienced greater reduction in PTSD symptoms (between group differences in post intervention, mean 12.49, 95% CI 6.82-18.17, p<0.001; d=1.22). Both groups experienced similar improvement in depression symptoms, PE-A group 24.32, 95% CI 18.18–30.46, P < 0.001, d = 3.18; difference in mean scores in the supportive counselling group 16.4, 95% CI 10.43–22.37, P < 0.001, d = 1.28). Both groups experienced similar improvement also in functioning (difference in mean scores in the PE-A group versus supportive counselling group −0.17, 95% CI −3.99 to 3.64, P = 0.93, d = 0.06)
Shangani et al, 2017, Kenya	Cohort	Community	Orphaned and vulnerable adolescents (OVA) (n=655)	14.0 (SD:2.4)	Unconditional cash transfers	The study assessed the impact of the government of Kenya unconditional cash transfers (CT). Through the programme, each household receive a cash payment of Kenya Shilling 1500 (around $20 USD) per month. This is equivalent to 20% of average monthly household expenditure in the region	Depressive symptoms- *Child Depression Inventory*<br>Anxiety- *Children's Manifest Anxiety Scale- Revised*<br>PTSD- *child PTSD checklist*	Post-intervention, OVA living in CT households were less likely to be anxious (OR 0.57, 95% CI 0.42, 0.78), less likely to have post-traumatic stress (OR 0.50, 95% CI 0.29, 0.89)<br>No impact of intervention on depressive symptoms
Ssewamala et al, 2017, Uganda	RCT	Community/ schools	AIDs-affected adolescents (n=1383)	12.8 (SD:1.2)	Savings-led family economic empowerment	***The Bridges study***<br>Participants received school material support i.e. food textbooks, and notebooks, counselling; financial literacy workshops on asset building, future planning, family microenterprise development, and protection from risks; mentorship; Child Savings Account (CSA)	Depressive symptoms- *Child Depression Inventory*<br>Hopelessness- *Beck Hopelessness Scale*	Intervention decreased adolescent's levels of depression by 0.25-0.29 standard deviation and levels of hopelessness by 0.18-0.23 standard deviation

Ssewamala et al, 2012, Uganda	RCT	Community/ schools	AIDS-orphaned adolescents (n=286)	Mean age= 13.7	Poverty reduction economic strengthening intervention	***Suubi (hope)-Uganda Project***<br>The intervention consisted of counselling; educational-related support e.g. textbooks; Microfinance intervention with three components; 1. Matched savings account; 2. Financial management classes; 3. Adult mentor to the children	Depressive symptoms- *Children's Depression Inventory*	Intervention group had a statistically reduction in depression over time (*B*= -0.34, *t* (256) = -.241, *p*= 0.02; 95% CI = -0.61, -0.06)<br>The control group's slope for the depression change over time was not statistically significant (*B*= -0.13, *t*(256) = -0.68, *p*= 0.50; 95% CI = -0.50, 0.24)
Thurman et al, South Africa, 201	Cluster RCT	Schools	Bereaved female adolescents (n=453)	13-17	Curriculum-based support group that incorporated indigenous stories and CBT components	***Abangane ("friends" in isiZulu)***<br>Sessions included: Introduction and relationship building; Identification and understanding feelings; Participants personal experiences of loss; Changes in life resulting from loss; Rituals and traditions surrounding loss; Coping skills; Looking to the future; Closure of the group	Adolescent grief- *Core Bereavement Items*<br>Depression- *Center for Epidemiological Studies–Depression Scale for Children (CES–DC)*	Intervention group had significantly lower scores for primary outcomes, including intrusive grief (p = 0·000, Cohen's d = –0·21), complicated grief (*p*= 0·015, *d*= –0·14), and depression (*p* =0·009, d = –0·21) relative to the waitlisted group after follow up
Thurman et al, 2018, South Africa	Pilot Pre–Post design,<br>* no control group	Community	Orphaned and vulnerable adolescents (n=105)	12-17	CBT- Family strengthening and HIV prevention intervention	***Let's Talk***<br>Participants meet in small closed-group 90-minute sessions led by a trained facilitator. The intervention includes CBT components; emphasizing goal-setting, challenging negative thoughts, and problem-solving skills, as well as adolescent-caregiver communication and condom and sexual refusal negotiation.	Adolescent and caregiver mental health- *Depression Anxiety Stress Scale*	Caregiver mental health issues (depression and anxiety) decreased by 22% (*p* = 0.007) and adolescent mental health by 26% (*p* = 0.004)
Thurman et al, 2017, South Africa	RCT	Community	Orphaned and vulnerable adolescents (n=489)	14-17	Interpersonal psychotherapy for groups (IPTG)	The intervention comprised of 16 weekly 90-minute group sessions focusing on interpersonal issues that can lead to depressive symptoms, such as grieving, disagreements, role changes, and relationship deficits. With a total of 15 IPTG groups led by 13 facilitators, participants were divided into gender groups with facilitators of the same gender.	Depressive symptoms- *Center for Epidemiologic Studies Depression Scale for Children (CES-DC)*	No significant treatment effect and moderation effect was found on the intervention in depressive symptomology (Coef = -0.838, *SE*= 1.129, *p*-value = 0.458)
Tol et al, 2014, Burundi	Cluster RCT	Schools	Children affected by armed conflict (n=329)	12.3 (SD:1.6)	CBT	***Classroom-based intervention (CBI)***<br>CBI included 15 sessions over 5 weeks implemented by locally trained facilitators. The intervention consisted of CBT techniques (psychoeducation, strengthening coping, and discussion of past traumatic events through drawing) and creative expressive elements (cooperative games, structured movement, music, drama, and dance)	PTSD- *Child Posttraumatic Symptom Scale*<br>Depression- *Depression Self-Rating Scale*<br>Anxiety- *Screen for Anxiety Related Emotional Disorder*<br>Hope- *Children's Hope Scale*<br>Hope- *Children's Hope Scale*	No main effects of the intervention were identified on mental health.<br>Mean changes (baseline to 3 months follow-up) for boys- PTSD (Mean = 5.68, *p*- value = 0.551), depression (Mean = 1.42, *p*- value = 0.149), hope (Mean = -1.03n, *p*- value = 0.586)<br>Mean changes (baseline to 3 months follow-up) for girls- PTSD (Mean = 5.85, *p*- value = 0.704), depression (Mean = -2.97, *p*- value = 0.292)<br>No effect sizes for anxiety

Ugwuanyi et al, 2020, Nigeria	RCT	Schools	Senior secondary school students (n=83)	18.2 (SD:4.7)	CBT	***CBT-music intervention program*** The intervention was implemented in a 12-week guided self-help group in which sessions were group-oriented. The infusion of music encompassed; listening to musical material; song writing, playing various music instruments, and using music as point of reference during group discussions. Sessions entailed discussions on causes of physics phobia, anxiety symptoms, and developing strategies on dealing with the anxiety.	Anxiety- *Generalized test anxiety inventory*	Post-treatment the effect of the intervention on reducing physics test anxiety was significant, F (1,80) =256.876, P=0.000, n^2_p =0.904, R^2 =0.956 and F (1,80) =243.873, P=0.000, n^2_p =0.904, R^2 =0.887.
Baird et al, 2013, Malawi	RCT	Schools	School girls (n= 3,796)	16.2 (SD:0.2)	Unconditional/ conditional cash transfers	Payment of school fees for participants in the conditional treatment arm but with a condition that they meet a monthly 80% school attendance rate and also received monthly cash transfers (between $1 and $5 per month). Attendance was never checked for recipients in the unconditional arm and they received their payments by simply presenting at the transfer locations each month; they also had their fees paid.	Psychological distress- *General Health Questionnaire*	The likelihood for suffering from psychological distress was 38% lower among girls who received unconditional cash transfers than controls<br>If the transfers were made conditional, the figure was 17 % lower compared to control.
Kilburn et al, 2019, South Africa	Cohort	Community/ Schools	High schools females (n= 2,533)	15 (IQR 14–17)	Conditional cash transfers	***HIV Prevention Trials Network (HPTN) 068 study*** Participants in the treatment arm received financial support in the form of monthly cash transfers(Approximately US$ 10 and US$ 20 for the girls and their parents respectively), conditional on regular school attendance (at least 80% of school days in the previous month) while the control arm received no transfers.	Depression- *Center for Epidemiological Studies Depression scale*<br>Hopelessness- *Hope scale*	The CCT improved psychosocial well-being including depression for young women from the poorest families but not the better off families (CES-D z-scores, -0.02, Hope z-scores, -0.02)
Walker et al, 2019, Nigeria	Cohort	Community	Displaced girls who had survived insurgency (n=50)	12-17	Skills acquisition program	***Science Village Gombe's skills acquisition program*** Three days a week for six months, the program included a mix of literacy training, skill acquisition, counselling, sports, and agricultural activities.	Psychological trauma- *interview sessions*	The results shows that acquiring skills alone does not inherently address psychological trauma, but can be embedded in a trauma treatment program
Mutiso et al, 2018, Kenya	Cohort	School	Orphans and vulnerable children in institutions of care (n=630)	15.1 (SD=2.0)	Psychoeducation	***Life skills education (LSE)*** Components entailed; critical and creative thinking, understanding mental health self-awareness and empathy, decision-making and problem solving, communication skills, interpersonal relations and coping skills such as relaxation techniques, and psychoeducation interventions.	Emotional and behavioural probles (externalizing and internalizing)- *The Youth Self Report*	A statistically significant reduction in externalizing, total problems and internalizing scores across the three groups (Group 1 achieved the greatest mean reduction of 7.12, while Experimental Group 2 had a mean reduction of 7.10 and controls had a 3.52 reduction)

Ssewamala et al, 2016, Uganda	RCT	Community	AIDS- orphaned adolescents (n=346)	13.4 (SD=1.24)	Family economic empowerment	***Suubi-Maka: "hope for families"*** The intervention entailed, providing adult mentors to children, financial management workshops, and promoting monetary savings for educational opportunities.	Hopelessness- *Beck Hopelessness Scale* Self-concept- *Tennessee Self-Concept Scale*	The mean Beck Hopelessness Scale score was 5.35 at baseline, and dropped to 3.26 at 24-month follow up, indicating a lower level of hopelessness. The average Tennessee Self-Concept Scale score was 78.4 at baseline, and rose to 84.2 at follow-up, corresponding to higher self-concept.
Burger et al, 2018 Tanzania	RCT	School	School going adolescents in grade 4-6	11-14	Psychoeducation program	***''ERSAE-Stress-Prosocial (ESPS)'' structured intervention*** Composed of sixteen 90-minute sessions divided into two sets of strategies: stress-reduction interventions and prosocial interventions including perspective-taking, empathy training, mindfulness and compassion-cultivating practices	Functional impairment- *self-report scale derived from Child Diagnostic Interview Schedule* Somatic complaints- *Diagnostic Predictive Scales* Hyperactivity- *Strengths and Difficulties Questionnaire* Anxiety/social difficulties- *Spence Anxiety scale*	The results suggested a significant overall impact of the intervention on all outcomes (time X measures X group; *Wilk's Lambda* = .32; *F* = 11.58; *df* = 27, 178; *p* < .001)
Kachingwe et al, 2021 Malawi	Cohort	Community	Young mothers(n=211)	17.6 (SD=7.5	Psychoeducation program	***Adolescent mothers project*** Interventions entailed providing a safe space for the young mothers. Sessions lasted 4 hours. Programs included adult learning, offered craft activities, small groups talk, and repeated opportunities to practice positive parenting.	Resilience- *Brief Resilience Scale* Self-esteem- *Rosenberg Self-Esteem Scale* Parental stress- *Parental Stress Scale*	No significant change in psychosocial well-being (*p* = 0.8823)
Westrhenen et al, 2019, South Africa	RCT	Community	Abused children (n=125)	10.0 (SD= 1.0-2.0)	Psychoeducation program	***Creative Arts in Psychotherapy (CAP)*** Intervention incorporated visual art, movement, dance, drama, music, and storytelling. The aim was to improve communications of emotions, interpersonal skills, resilience, and reduce trauma stress	PTSD- *Child PTSD Checklist* Behavioural problems (externalizing and internalizing)- *Child Behaviour Checklist* Posttraumatic growth- *Posttraumatic Growth Inventory*	Hyper arousal symptoms (d = 0.61) and avoidance symptoms (d = 0.41) decreased more in the treatment group compared to the control group. No impact on behavioural problems and posttraumatic growth
McMullen et al, 2018, Uganda	RCT	Schools	Students (n=620)	13-18	Life skills development	***The Living Well Programme*** It incorporates 24 lessons plans, with 6 lessons in each of four overarching themes: 1) Living Well with Ourselves and Others; 2) Living Well with Worry and Stress; 3) Living Well with Life's Issues; and 4) Living Well in the Future.	Self-belief- *The General Self-Efficacy Scale* Depression/anxiety-like symptoms- *The Youth Psychosocial Assessment Instrument*	Intervention group had a significant increase in general self-efficacy, $F(1,167) = 20.10$, $p < .001$, $\eta 2 = 0.107$, significant reductions in internalising problems (depression/anxiety-like symptoms), $F(1,167) = 11.14$, $p = .001$, $\eta 2 = 0.063$, and an increase in overall

						Modules aims to promote mental health resilience, develop communication skills, make good choices, and develop life skills.	Connectedness- *The Hemingway Measure of Adolescent Connectedness*	‘Connectedness’ F(1,167) = 15.00, p < .001, η2 = 0.082, when compared to the control group
Murray et al, 2013, Zambia	Cohort	Community	Orphans and vulnerable children (n=94)	12.9 (5-18)	TF-CBT	Intervention was conducted through weekly TF-CBT sessions lasting 1–2 h over an average of 11 weeks (range 8–23). Sessions included a mix of the child alone, a caregiver(s) alone, and the family members together.	PTSD- *The Post-Traumatic Stress Disorder- Reaction Index*	Mean PTSD score after treatment (27.6) was significantly lower than the average pre-treatment score (67.7, p < 0.0001)
Dow et al, 2020, Tanzania	RCT	Clinics	Young people living with HIV (n=128)	Mean age= 18.1	CBT	***Sauti ya Vijana (The Voice of Youth)*** The intervention involved 10 group sessions, in which was firstly conducted in individual session to discuss participants past memories, and to prepare them on group discussions. The other interventions entailed inviting caregivers to share experiences altogether.	Depression- *The Patient Health Questionnaire* Emotional and behavioural symptoms- *The strengths and Difficulties Questionnaire* PTSD- *UCLA Post Traumatic Stress Symptoms Exposure Screener and Reaction Index survey*	A reported reduction of: 4 points of PHQ 9 score, 3.9 of the SDQ score, and no change in the UCLA trauma score in the intervention group
Ssewamala, 2021, Uganda	RCT	Schools	Adolescents orphaned by HIV/AIDS (n=896)	12(SD=1.2)	Economic support	***Bridges study*** Participants received school lunches, scholastic materials including textbooks and notebooks, and counselling. Components entailed; workshops on finance literacy, mentorships, and matched financing account. Participants in the Bridges condition received a 1:1 savings match rate, whereas participants in the Bridges PLUS, condition received a 2:1 savings matching rate.	Depressive symptoms- *Children's Depression Inventory* Hopelessness- *Beck Hopelessness Scale*	Bridges and Bridges PLUS participants reported higher physical health scores, lower depressive symptoms, and higher self-concept and self-efficacy
Fine et al, 2021, Burundi	Cluster RCT	Community	Young refugee adolescents (n=86)	12.3(SD=1.5)	CBT	***EASE intervention*** Intervention entailed 7 weekly group sessions. Components included; psychoeducation, stress management, behavioural activation, problem solving and relapse prevention. ETAU entailed psychoeducation sessions which focused on strategies for self-care, and about psychosocial support.	Psychosocial distress- *African Youth Psychosocial Assessment* *PTSD*- Child PTSD Symptom Scale	There was a statistically significant decrease in psychological distress in both the EASE group (mean change = −6.7, *p* < 0.001) and the ETAU group (mean change = −4.3, *p* = 0.02). This change was driven by significant decreases in internalizing symptoms among both groups (EASE mean change = −5.5, *p* < 0.001; ETAU mean change = −4.5, *p* = 0.004) as well as somatic complaints in the EASE group (mean change = −1.3, *p* < 0.001).

Kane, et al, 2016, Zambia	RCT	Community	Orphans and vulnerable children (n=257)	13.7(SD=2.9)	TF-CBT	There were 9 core components including psychoeducation, parenting skills, relaxation skills, affective modulation skills, cognitive coping skills, trauma narration and processing, in vivo mastery of trauma reminders (live exposure), conjoint session (with caregiver), and enhancing safety skills.<br>The TAU services included education, support groups, primary healthcare services, nutrition education, HIV/AIDS counselling, and/ or psychosocial counselling.	PTSD- *Post-Traumatic Stress Disorder Reaction Index*<br>Functional impairment- *locally developed gender-specific scale*	The effect sizes of TF-CBT for the trauma symptom and functioning outcomes were 2.39 and 0.34, respectively.
Chory et al, 2021, Kenya	Cohort	Community	Adolescents living with HIV (n=30)	Mean age= 15.5	Digital intervention	Intervention entailed the delivery of individual counselling support through the WhatsApp platform	Depression- *Patient Health Questionnaire, The Hopkins Symptom Checklist for Depression*	There was no discernible change over time in respondents' scores on depression assessments
Murray et al, 2015, Zambia	RCT	Community/schools	Children experienced at least one traumatic event (n=257)	13.7 (SD=	TF-CBT	TF-CBT intervention included the following components; psychoeducation, parenting skills, relaxation, affective modulation, cognitive coping, trauma narrative, in vivo exposure, enhancing safety skills, and conjoint session.<br>TAU included the usual support adolescents gets in the community outreaches, and clinics	PTSD- *PTSD-RI*	The mean item change in trauma symptom score at follow-up was −1.54 (95% CI, −1.81 to −1.27), a reduction of 81.9%, for the TF-CBT group and −0.37 (95% CI, −0.57 to −0.17), a reduction of 21.1%, for the TAU group. The mean item change for functioning was −0.76 (95% CI, −0.98 to −0.54), a reduction of 89.4%, and −0.54 (95% CI, −0.80 to −0.29), a reduction of 68.3%, for the TF-CBT and TAU groups, respectively.
Talbot et al, 2013, Rwanda	Cohort	Community	Orphans (n=120)	18(15-25)	Counselling	Mental health components included; adult mentorship, group and individual counselling.<br>HIV education program consisted of group discussion, and presentations on sexuality, and HIV and strategies to prevent its transmission	Psychological trauma symptoms- *Post-Traumatic Stress Disorder Check List*<br>Social functioning- *Acceptance and Action Questionnaire*	Increased use of counseling services was associated with a decline in trauma symptoms over time (-0.40; p = 0.032),

CBI: classroombased intervention; **CBT**: cognitive behavioral therapy; **CCT**: conditional cash transfers; **CI**: confidence intervals; **CSA**: child savings account; **CT**: cash transfers; FSI: family strengthening intervention; **HIV/AIDs**: human immunodeficiency virus, acquired immunodeficiency syndrome; **IPT**: interpersonal psychotherapy; **IPTG**: interpersonal psychotherapy for groups; **OVA**: orphaned and vulnerable adolescents; **PE-A**: prolonged exposure therapy; **PTSD**: post-traumatic stress disorder; **RCT**: randomized controlled trial; **SD**: standard deviation; SSIs: computerized single-session interventions; **TAU**: treatment as usual; **TF-CBT**: trauma focused cognitive behavioral therapy; **UC**: usual care; **UCT**: unconditional cash transfer; US$: United States dollar; **YRI**: youth readiness intervention; **AOR:**adjusted odds ratio; TG: treatment group; CG: control group; MS: mean scores; CP: creative play; ASD: Autism Spectrum Disorder
